# Supplementary material for: Closed-loop neuromodulation restores network connectivity and motor control after spinal cord injury
Source: eLife. 2018 Mar 13;7:e32058. doi: 10.7554/eLife.32058 (PMC5849415; doi:10.7554/eLife.32058)
Supplement: Supplementary file 1. — The values in the table below represent the average peak pull force for all rats included in the study at each week during therapy. Empty cells correspond to weeks in which data was not collected, based on the design of the experiment. All rat IDs are consistent for individual subjects throughout Supplementary file 1–4. [file elife-32058-supp1.docx]

**Supplementary File 1: Volitional Forelimb Strength Behavioral Data**

The values in the table below represent the average peak pull force for all rats included in the study at each week during therapy. Empty cells correspond to weeks in which data was not collected, based on the design of the experiment. All rat IDs are consistent for individual subjects throughout Supplementary Files 1-4.

| **ID** | **SCI** | **Group** | **PRE** | **Wk 6** | **Wk 7** | **Wk 8** | **Wk 9** | **Wk 10** | **Wk 11** | **Wk 12** | **Wk 13** | **Wk 14** |
| --- | --- | --- | --- | --- | --- | --- | --- | --- | --- | --- | --- | --- |
| Rat 001 | Unilateral | Rehab alone | 138.0 | 46.1 | 55.0 | 69.7 | 75.1 | 79.2 | 86.6 | 95.7 |  |  |
| Rat 002 | Unilateral | Rehab alone | 140.8 | 81.4 | 75.4 | 100.5 | 105.7 | 109.5 | 99.8 | 88.2 |  |  |
| Rat 003 | Unilateral | Rehab alone | 140.0 | 63.4 | 84.7 | 105.9 | 103.5 | 123.2 | 121.6 | 134.4 |  |  |
| Rat 004 | Unilateral | Rehab alone | 172.2 | 63.0 | 83.2 | 91.6 | 93.0 | 93.9 | 86.0 | 89.0 |  |  |
| Rat 005 | Unilateral | Rehab alone | 161.1 | 87.7 | 90.3 | 105.4 | 124.2 | 124.6 | 113.3 | 112.6 |  |  |
| Rat 006 | Unilateral | Rehab alone | 181.2 | 90.7 | 100.5 | 115.8 | 122.9 | 123.1 | 120.7 | 123.9 |  |  |
| Rat 007 | Unilateral | Rehab alone | 146.9 | 49.4 | 54.9 | 69.1 | 81.6 | 83.2 | 101.1 | 88.6 |  |  |
| Rat 008 | Unilateral | Rehab alone | 163.0 | 52.3 | 62.4 | 78.0 | 101.0 | 113.5 | 101.4 | 100.9 |  |  |
| Rat 009 | Unilateral | Rehab alone | 143.1 | 56.1 | 56.6 | 78.9 | 78.6 | 84.1 | 93.2 | 111.1 |  |  |
| Rat 010 | Unilateral | Rehab alone | 168.0 | 76.3 | 81.2 | 93.3 | 101.0 | 106.9 | 87.1 | 65.6 |  |  |
| Rat 011 | Unilateral | Rehab alone | 149.2 | 54.6 | 72.2 | 79.0 | 101.9 | 116.3 | 94.4 | 100.8 |  |  |
| Rat 012 | Unilateral | Rehab alone | 154.3 | 69.2 | 85.4 | 99.0 | 96.6 | 112.4 | 120.7 | 117.7 |  |  |
| Rat 013 | Unilateral | Rehab alone | 129.3 | 48.3 | 61.2 | 77.3 | 82.4 | 88.4 | 85.3 | 89.1 |  |  |
| Rat 014 | Unilateral | Rehab alone | 148.7 | 38.1 | 76.3 | 77.1 | 85.1 | 85.2 | 79.8 | 81.7 |  |  |
| Rat 015 | Unilateral | Rehab alone | 140.8 | 36.5 | 52.3 | 50.6 | 63.3 | 58.2 | 62.7 | 62.9 |  |  |
| Rat 016 | Unilateral | Rehab alone | 158.1 | 47.1 | 83.9 | 87.5 | 97.7 | 114.4 | 106.5 | 88.9 |  |  |
| Rat 017 | Unilateral | Rehab alone | 147.4 | 46.8 | 60.7 | 62.9 | 67.4 | 65.4 | 71.4 | 75.2 |  |  |
| Rat 018 | Unilateral | Top 50% CLV | 153.9 | 73.3 | 73.9 | 87.4 | 105.0 | 115.6 | 125.2 | 118.4 |  |  |
| Rat 019 | Unilateral | Top 50% CLV | 162.2 | 79.3 | 65.7 | 89.7 | 94.5 | 122.7 | 134.4 | 140.8 |  |  |
| Rat 020 | Unilateral | Top 50% CLV | 143.2 | 99.3 | 96.7 | 96.1 | 86.9 | 95.1 | 94.4 | 106.0 |  |  |
| Rat 021 | Unilateral | Top 50% CLV | 161.7 | 97.0 | 127.4 | 136.8 | 120.6 | 117.6 | 133.7 | 139.0 |  |  |
| Rat 022 | Unilateral | Top 50% CLV | 155.2 | 77.3 | 109.7 | 116.9 | 130.7 | 131.7 | 106.3 | 108.2 |  |  |
| Rat 023 | Unilateral | Top 50% CLV | 182.8 | 93.1 | 120.3 | 133.5 | 131.9 | 131.6 | 129.9 | 137.5 |  |  |
| Rat 024 | Unilateral | Top 50% CLV | 155.8 | 53.0 | 75.1 | 94.2 | 102.8 | 115.3 | 121.0 | 117.9 |  |  |
| Rat 025 | Unilateral | Top 50% CLV | 152.7 | 42.9 | 56.0 | 76.7 | 117.2 | 132.6 | 126.6 | 136.0 |  |  |
| Rat 026 | Unilateral | Top 50% CLV | 149.7 | 33.4 | 66.9 | 82.3 | 109.4 | 134.3 | 127.6 | 118.1 |  |  |
| Rat 027 | Unilateral | Top 50% CLV | 146.6 | 81.2 | 84.5 | 91.7 | 150.2 | 144.5 | 136.9 | 138.4 |  |  |
| Rat 028 | Unilateral | Top 50% CLV | 177.7 | 44.2 | 83.2 | 119.2 | 129.0 | 141.8 | 143.3 | 144.1 |  |  |
| Rat 029 | Unilateral | Top 50% CLV | 157.0 | 44.0 | 70.3 | 85.8 | 117.2 | 127.9 | 128.2 | 129.9 |  |  |
| Rat 030 | Unilateral | Top 50% CLV | 148.9 | 43.1 | 93.0 | 110.0 | 113.4 | 111.6 | 111.7 | 110.6 |  |  |
| Rat 031 | Unilateral | Top 50% CLV | 163.1 | 66.7 | 54.7 | 78.2 | 94.6 | 113.5 | 98.6 | 95.0 |  |  |
| Rat 030 | Bilateral | Rehab alone | 156.1 |  |  | 45.5 | 49.0 | 60.6 | 68.8 | 61.4 | 64.0 | 72.6 |
| Rat 031 | Bilateral | Rehab alone | 155.1 |  |  | 80.6 | 84.8 | 90.1 | 89.2 | 101.5 | 98.0 | 99.1 |
| Rat 032 | Bilateral | Rehab alone | 161.7 |  |  | 79.5 | 100.6 | 117.9 | 116.6 | 133.5 | 129.7 | 119.4 |
| Rat 033 | Bilateral | Rehab alone | 192.4 |  |  | 84.3 | 83.7 | 93.6 | 108.3 | 116.6 | 121.8 | 119.9 |
| Rat 034 | Bilateral | Rehab alone | 155.5 |  |  | 49.7 | 90.9 | 91.5 | 92.3 | 98.0 | 109.9 | 109.5 |
| Rat 035 | Bilateral | Rehab alone | 148.3 |  |  | 102.4 | 105.2 | 109.0 | 135.2 | 134.2 | 126.8 | 141.6 |
| Rat 036 | Bilateral | Rehab alone | 144.1 |  |  | 23.0 | 38.2 | 39.3 | 36.1 | 43.9 | 38.9 | 48.3 |
| Rat 037 | Bilateral | Rehab alone | 150.4 |  |  | 74.8 | 53.4 | 71.1 | 69.1 | 51.6 | 51.4 | 62.6 |
| Rat 038 | Bilateral | Rehab alone | 145.9 |  |  | 31.0 | 40.8 | 51.5 | 55.1 | 53.7 | 41.8 | 59.8 |
| Rat 039 | Bilateral | Rehab alone | 146.3 |  |  | 30.3 | 34.5 | 35.2 | 41.6 | 48.1 | 43.7 | 26.9 |
| Rat 040 | Bilateral | Top 50% CLV | 152.0 |  |  | 67.9 | 83.6 | 90.7 | 92.0 | 93.8 | 101.9 | 104.5 |
| Rat 041 | Bilateral | Top 50% CLV | 150.8 |  |  | 67.4 | 70.1 | 104.3 | 100.6 | 123.2 | 129.3 | 134.8 |
| Rat 042 | Bilateral | Top 50% CLV | 138.6 |  |  | 49.1 | 82.7 | 108.4 | 122.6 | 125.1 | 117.9 | 133.7 |
| Rat 043 | Bilateral | Top 50% CLV | 162.9 |  |  | 77.9 | 86.5 | 99.1 | 104.4 | 106.6 | 93.7 | 104.7 |
| Rat 044 | Bilateral | Top 50% CLV | 157.6 |  |  | 76.6 | 139.8 | 140.7 | 147.7 | 145.6 | 140.3 | 135.9 |
| Rat 045 | Bilateral | Top 50% CLV | 148.5 |  |  | 91.2 | 99.1 | 129.7 | 136.4 | 125.7 | 135.2 | 125.1 |
| Rat 046 | Bilateral | Top 50% CLV | 183.3 |  |  | 84.9 | 126.0 | 126.0 | 119.8 | 123.9 | 130.6 | 136.3 |
| Rat 047 | Bilateral | Top 50% CLV | 159.2 |  |  | 78.9 | 118.5 | 133.1 | 138.8 | 133.7 | 126.1 | 115.8 |
| Rat 048 | Bilateral | Top 50% CLV | 149.0 |  |  | 60.6 | 115.0 | 131.2 | 130.1 | 133.3 | 138.9 | 134.2 |
| Rat 049 | Bilateral | Top 50% CLV | 170.5 |  |  | 46.3 | 74.5 | 96.8 | 91.3 | 98.6 | 104.3 | 100.9 |
| Rat 050 | Bilateral | Top 50% CLV | 155.8 |  |  | 55.8 | 77.2 | 86.8 | 79.0 | 108.7 | 127.8 | 134.8 |
| Rat 049 | Unilateral | Top 20% CLV | 174.5 | 63.2 | 86.5 | 122.1 | 125.3 | 138.4 | 149.4 | 121.4 |  |  |
| Rat 050 | Unilateral | Top 20% CLV | 167.6 | 26.8 | 52.9 | 80.3 | 102.6 | 114.1 | 110.3 | 120.6 |  |  |
| Rat 051 | Unilateral | Top 20% CLV | 176.7 | 17.8 | 76.6 | 71.6 | 90.4 | 133.7 | 151.8 | 148.2 |  |  |
| Rat 052 | Unilateral | Top 20% CLV | 159.6 | 38.3 | 81.9 | 147.3 | 159.3 | 145.1 | 154.7 | 158.7 |  |  |
| Rat 053 | Unilateral | Top 20% CLV | 174.6 | 28.8 | 47.6 | 50.9 | 88.1 | 93.1 | 118.3 | 127.8 |  |  |
| Rat 054 | Unilateral | Top 20% CLV | 142.3 | 19.7 | 51.7 | 69.9 | 77.0 | 80.9 | 91.4 | 90.6 |  |  |
| Rat 055 | Unilateral | Top 20% CLV | 150.1 | 53.8 | 89.0 | 118.9 | 129.2 | 137.0 | 133.7 | 144.6 |  |  |
| Rat 056 | Unilateral | Top 20% CLV | 145.6 | 35.3 | 54.4 | 112.1 | 125.8 | 135.2 | 140.0 | 133.8 |  |  |
| Rat 057 | Unilateral | Top 20% CLV | 168.6 | 30.1 | 62.8 | 90.1 | 88.8 | 102.1 | 108.3 | 124.0 |  |  |
| Rat 058 | Unilateral | Top 20% CLV | 142.0 | 41.8 | 48.2 | 53.2 | 69.2 | 95.5 | 112.7 | 129.1 |  |  |
| Rat 059 | Unilateral | Top 20% CLV | 152.6 | 47.4 | 41.0 | 77.7 | 107.7 | 136.8 | 159.1 | 160.2 |  |  |
| Rat 060 | Unilateral | Top 20% CLV | 143.4 | 30.2 | 38.6 | 56.7 | 76.8 | 95.9 | 115.8 | 119.2 |  |  |
| Rat 061 | Unilateral | Top 20% CLV | 171.4 | 34.4 | 51.1 | 69.3 | 79.6 | 90.2 | 91.5 | 96.4 |  |  |
| Rat 062 | Unilateral | Bottom 20% CLV | 158.1 | 15.8 | 41.2 | 78.1 | 60.1 | 96.3 | 110.9 | 121.8 |  |  |
| Rat 063 | Unilateral | Bottom 20% CLV | 167.0 | 51.3 | 59.4 | 87.5 | 95.5 | 108.6 | 96.9 | 107.0 |  |  |
| Rat 064 | Unilateral | Bottom 20% CLV | 159.4 | 34.4 | 42.8 | 62.5 | 60.6 | 89.6 | 113.8 | 115.4 |  |  |
| Rat 065 | Unilateral | Bottom 20% CLV | 144.3 | 18.9 | 54.1 | 85.7 | 95.6 | 98.0 | 97.6 | 100.5 |  |  |
| Rat 066 | Unilateral | Bottom 20% CLV | 134.3 | 26.5 | 56.8 | 59.9 | 64.0 | 80.8 | 53.3 | 76.4 |  |  |
| Rat 067 | Unilateral | Bottom 20% CLV | 160.3 | 31.6 | 63.7 | 102.5 | 119.3 | 107.4 | 132.5 | 125.7 |  |  |
| Rat 068 | Unilateral | Bottom 20% CLV | 156.1 | 45.4 | 30.8 | 41.4 | 53.7 | 63.6 | 76.9 | 94.8 |  |  |
| Rat 069 | Unilateral | Bottom 20% CLV | 153.2 | 38.7 | 48.6 | 78.9 | 87.6 | 82.4 | 88.9 | 85.9 |  |  |
| Rat 070 | Unilateral | Rehab alone | 159.1 | 32.2 | 63.7 | 68.3 | 83.3 | 104.4 | 86.6 | 79.1 |  |  |
| Rat 071 | Unilateral | Rehab alone | 179.4 | 23.5 | 50.9 | 71.2 | 112.9 | 127.1 | 119.1 | 130.0 |  |  |
| Rat 072 | Unilateral | Rehab alone | 142.2 | 43.2 | 63.4 | 83.7 | 93.3 | 111.4 | 103.3 | 121.0 |  |  |
| Rat 073 | Unilateral | Rehab alone | 163.8 | 28.7 | 32.2 | 50.0 | 56.7 | 84.1 | 107.9 | 104.7 |  |  |
| Rat 074 | Unilateral | Rehab alone | 133.7 | 27.0 | 55.5 | 62.1 | 69.1 | 76.7 | 77.2 | 85.5 |  |  |
| Rat 075 | Unilateral | Rehab alone | 150.5 | 42.0 | 74.8 | 94.9 | 123.6 | 133.0 | 128.8 | 121.0 |  |  |
| Rat 076 | Unilateral | Rehab alone | 143.8 | 47.9 | 55.4 | 76.5 | 86.4 | 97.1 | 100.4 | 79.8 |  |  |
| Rat 077 | Unilateral | Rehab alone | 147.5 | 52.0 | 76.0 | 98.1 | 115.4 | 118.7 | 111.4 | 67.2 |  |  |
| Rat 078 | Unilateral | Rehab alone | 153.8 | 25.1 | 29.2 | 43.0 | 44.7 | 63.9 | 69.9 | 58.6 |  |  |
