# Supplementary material for: Closed-loop neuromodulation restores network connectivity and motor control after spinal cord injury
Source: eLife. 2018 Mar 13;7:e32058. doi: 10.7554/eLife.32058 (PMC5849415; doi:10.7554/eLife.32058)
Supplement: Supplementary file 2. — The values in the table below represent the area of motor cortex in mm (Levy et al., 2016) evoking movements in each of the categories. All rat IDs are consistent for individual subjects throughout Supplementary file 1–4. [file elife-32058-supp2.docx]

**Supplementary File 2: Intracortical Microstimulation Data**

The values in the table below represent the area of motor cortex in mm^2^ evoking movements in each of the categories. All rat IDs are consistent for individual subjects throughout Supplementary Files 1-4.

| **ID** | **SCI** | **Group** | **Total Area** | **Grasp** | **Wrist** | **Elbow** | **Shoulder** | **Vibrissa** | **Neck** | **Jaw** | **Hindlimb** | **Trunk** |
| --- | --- | --- | --- | --- | --- | --- | --- | --- | --- | --- | --- | --- |
| Rat 001 | Unilateral | Rehab alone | 10.5 | 0.5 | 0.5 | 2 | 0.75 | 3.25 | 1.75 | 0 | 1.75 | 0 |
| Rat 004 | Unilateral | Rehab alone | 6 | 0 | 3.375 | 0.375 | 0 | 2 | 0.25 | 0 | 0 | 0 |
| Rat 005 | Unilateral | Rehab alone | 10.5 | 0.75 | 0.125 | 4.375 | 0 | 4 | 1 | 0 | 0.25 | 0 |
| Rat 006 | Unilateral | Rehab alone | 9.75 | 0 | 3.5 | 2.5 | 0 | 0.75 | 0.5 | 0 | 2.5 | 0 |
| Rat 007 | Unilateral | Rehab alone | 6.5 | 0.5 | 0.125 | 2.125 | 0 | 1.25 | 1.25 | 0 | 0.75 | 0.5 |
| Rat 008 | Unilateral | Rehab alone | 7.5 | 0.375 | 2.25 | 1.625 | 0.75 | 2 | 0 | 0 | 0.5 | 0 |
| Rat 020 | Unilateral | Top 50% CLV | 14.5 | 0.5 | 4.75 | 1.75 | 0 | 3.5 | 0.25 | 0 | 3.75 | 0 |
| Rat 021 | Unilateral | Top 50% CLV | 9.75 | 0.875 | 4.25 | 0.875 | 0 | 1.25 | 1 | 0 | 1.5 | 0 |
| Rat 022 | Unilateral | Top 50% CLV | 7 | 1 | 0.5 | 1.5 | 0.5 | 2.75 | 0.25 | 0 | 0.5 | 0 |
| Rat 023 | Unilateral | Top 50% CLV | 6.75 | 2.5 | 0.25 | 1 | 0 | 1.75 | 0.25 | 0 | 1 | 0 |
| Rat 024 | Unilateral | Top 50% CLV | 3.75 | 3.25 | 0 | 0 | 0 | 0.5 | 0 | 0 | 0 | 0 |
| Rat 025 | Unilateral | Top 50% CLV | 6 | 3.25 | 0.25 | 0.5 | 0.25 | 1.5 | 0 | 0 | 0.25 | 0 |
| Rat 030 | Bilateral | Rehab alone | 11.75 | 0.625 | 0 | 3.75 | 0.625 | 2.5 | 1.75 | 2.5 | 0 | 0 |
| Rat 031 | Bilateral | Rehab alone | 7 | 0 | 0 | 4.75 | 0 | 2.25 | 0 | 0 | 0 | 0 |
| Rat 032 | Bilateral | Rehab alone | 13.25 | 0.625 | 0 | 5.125 | 0 | 4.25 | 1.5 | 1.75 | 0 | 0 |
| Rat 033 | Bilateral | Rehab alone | 13 | 0 | 0 | 5.25 | 0 | 4 | 2 | 1.75 | 0 | 0 |
| Rat 034 | Bilateral | Rehab alone | 10 | 3.25 | 0.625 | 2.875 | 0 | 1.25 | 0.5 | 1.5 | 0 | 0 |
| Rat 035 | Bilateral | Rehab alone | 13.75 | 1.5 | 0 | 3.25 | 0.25 | 6 | 1.75 | 1 | 0 | 0 |
| Rat 036 | Bilateral | Rehab alone | 10.75 | 2 | 0 | 2.5 | 0 | 4.75 | 1 | 0.5 | 0 | 0 |
| Rat 041 | Bilateral | Top 50% CLV | 13 | 0 | 0.25 | 3.25 | 0 | 8.25 | 0.5 | 0.75 | 0 | 0 |
| Rat 042 | Bilateral | Top 50% CLV | 3.75 | 0.125 | 0.125 | 2 | 0 | 0.75 | 0.75 | 0 | 0 | 0 |
| Rat 043 | Bilateral | Top 50% CLV | 11 | 2.875 | 0 | 4.625 | 0.5 | 2 | 0.25 | 0.75 | 0 | 0 |
| Rat 044 | Bilateral | Top 50% CLV | 11.75 | 0.125 | 0 | 6.875 | 0 | 2 | 2.25 | 0.5 | 0 | 0 |
| Rat 045 | Bilateral | Top 50% CLV | 8 | 1.375 | 0.25 | 4.125 | 0 | 1.75 | 0 | 0.5 | 0 | 0 |
| Rat 046 | Bilateral | Top 50% CLV | 6.5 | 0.25 | 0 | 2.75 | 0 | 2.5 | 0.5 | 0.5 | 0 | 0 |
| Rat 049 | Bilateral | Top 50% CLV | 10 | 1.5 | 0.25 | 2.5 | 0.75 | 3.25 | 1.75 | 0 | 0 | 0 |
| Rat 079 | None | Control | 9.75 | 0.25 | 1.25 | 2.75 | 0 | 4.25 | 0.5 | 0 | 0.75 | 0 |
| Rat 080 | None | Control | 7.75 | 0.25 | 0 | 2.75 | 0 | 3 | 0.5 | 1 | 0.25 | 0 |
| Rat 081 | None | Control | 5.25 | 0.625 | 0.375 | 3.25 | 0 | 0.25 | 0 | 0 | 0.75 | 0 |
| Rat 082 | None | Control | 4.75 | 0.875 | 0.25 | 2.625 | 0 | 0.25 | 0.75 | 0 | 0 | 0 |
| Rat 083 | None | Control | 10 | 0.25 | 1.5 | 2.625 | 0.125 | 2.5 | 1.5 | 0 | 1.25 | 0.25 |
| Rat 084 | None | Control | 5.25 | 0.75 | 0 | 1.875 | 0.625 | 1 | 0.75 | 0 | 0.25 | 0 |
| Rat 085 | None | Control | 5.5 | 1.25 | 1.25 | 0.5 | 0 | 1.25 | 0 | 0 | 1.25 | 0 |
