# Supplementary material for: Closed-loop neuromodulation restores network connectivity and motor control after spinal cord injury
Source: eLife. 2018 Mar 13;7:e32058. doi: 10.7554/eLife.32058 (PMC5849415; doi:10.7554/eLife.32058)
Supplement: Supplementary file 3. — The values in the table below represent the total number of labelled eGFP+ neurons in each of the ROIs below. For rostrocaudal distribution of labeled neurons, see Figure 2—figure supplement 3 and Figure 4—figure supplement 5. All rat IDs are consistent for individual subjects throughout Supplementary file 1–4. [file elife-32058-supp3.docx]

**Supplementary File 3: Pseudorabies Virus Labeling Data**

The values in the table below represent the total number of labelled eGFP+ neurons in each of the ROIs below. For rostrocaudal distribution of labeled neurons, see Fig. 2-Figure Supplement 5 and Fig. 4-Figure Supplement 3. All rat IDs are consistent for individual subjects throughout Supplementary Files 1-4.

| **ID** | **SCI** | **Group** | **Left Spinal Motor** | **Right Spinal Motor** | **Left Motor Cortex** | **Right Motor Cortex** | **Left Red Nucleus** | **Right Red Nucleus** | **Left C3/4 Propriospinal Neurons** | **Right C3/4 Propriospinal Neurons** |
| --- | --- | --- | --- | --- | --- | --- | --- | --- | --- | --- |
| Rat 009 | Unilateral | Rehab alone | 0 | 14 | 16 | 26 | 46 | 44 | 70 | 68 |
| Rat 010 | Unilateral | Rehab alone | 2 | 17 | 107 | 87 | 15 | 124 | 139 | 143 |
| Rat 011 | Unilateral | Rehab alone | 0 | 6 | 55 | 28 | 20 | 46 | 178 | 103 |
| Rat 012 | Unilateral | Rehab alone | 0 | 18 | 34 | 45 | 64 | 61 | 229 | 184 |
| Rat 013 | Unilateral | Rehab alone | 1 | 11 | 49 | 17 | 16 | 37 | 105 | 70 |
| Rat 014 | Unilateral | Rehab alone | 1 | 14 | 32 | 32 | 6 | 97 | 102 | 84 |
| Rat 026 | Unilateral | Top 50% CLV | 3 | 9 | 245 | 293 | 22 | 68 | 147 | 109 |
| Rat 027 | Unilateral | Top 50% CLV | 1 | 5 | 87 | 52 | 0 | 46 | 148 | 100 |
| Rat 028 | Unilateral | Top 50% CLV | 0 | 11 | 193 | 29 | 37 | 46 | 73 | 50 |
| Rat 029 | Unilateral | Top 50% CLV | 0 | 21 | 469 | 178 | 43 | 86 | 202 | 147 |
| Rat 031 | Unilateral | Top 50% CLV | 0 | 11 | 212 | 75 | ‡ | ‡ | 58 | 56 |
| Rat 037 | Bilateral | Rehab alone | 3 | 12 | 43 | 32 | 59 | 10 | 130 | 144 |
| Rat 038 | Bilateral | Rehab alone | 0 | 15 | 46 | 33 | 77 | 8 | 56 | 45 |
| Rat 039 | Bilateral | Rehab alone | 0 | 11 | 50 | 22 | 80 | 4 | 100 | 63 |
| Rat 047 | Bilateral | Top 50% CLV | 0 | 15 | 53 | 58 | 154 | 8 | 166 | 298 |
| Rat 048 | Bilateral | Top 50% CLV | 1 | 9 | 150 | 67 | 148 | 35 | 75 | 111 |
| Rat 050 | Bilateral | Top 50% CLV | 0 | 10 | 24 | 57 | 132 | 8 | 122 | 173 |
| Rat 086 | None | Control | 0 | 26 | 475 | 222 | 250 | 26 | 377 | 526 |
| Rat 087 | None | Control | 2 | 14 | 114 | 50 | 257 | 131 | 142 | 182 |
| Rat 088 | None | Control | 3 | 23 | 798 | 361 | 280 | 92 | 280 | 419 |
| Rat 089 | None | Control | 0 | 22 | 267 | 100 | 226 | 33 | 229 | 351 |
| Rat 090 | None | Control | 2 | 26 | 192 | 38 | 264 | 61 | 97 | 78 |

*‡ denotes missing data due to technical issues*
