# Supplementary material for: Closed-loop neuromodulation restores network connectivity and motor control after spinal cord injury
Source: eLife. 2018 Mar 13;7:e32058. doi: 10.7554/eLife.32058 (PMC5849415; doi:10.7554/eLife.32058)
Supplement: Supplementary file 4. — The values in the table below represent the estimated percent damage to gray matter and white matter tracts. All rat IDs are consistent for individual subjects throughout Supplementary file 1–4. [file elife-32058-supp4.docx]

**Supplementary File 4: Lesion Metrics**

The values in the table below represent the estimated percent damage to gray matter and white matter tracts. All rat IDs are consistent for individual subjects throughout Supplementary Files 1-4.

|  |  |  | **Right Hemicord** | | | | **Left Hemicord** | | | |
| --- | --- | --- | --- | --- | --- | --- | --- | --- | --- | --- |
| **ID** | **SCI** | **Group** | **Gray Matter** | **Corticospinal Tract** | **Rubrospinal Tract** | **PropriospinalTract** | **Gray Matter** | **Corticospinal Tract** | **Rubrospinal Tract** | **PropriospinalTract** |
| Rat 001 | Unilateral | Rehab alone | 75 | 3.4 | 100 | 80 | 0 | 0 | 0 | 0 |
| Rat 002 | Unilateral | Rehab alone | 77.5 | 3.4 | 100 | 62.5 | 0 | 0 | 0 | 0 |
| Rat 003 | Unilateral | Rehab alone | 70 | 3.4 | 100 | 92.5 | 0 | 0 | 0 | 0 |
| Rat 005 | Unilateral | Rehab alone | 62.5 | 3.4 | 100 | 85 | 0 | 0 | 0 | 0 |
| Rat 006 | Unilateral | Rehab alone | 55 | 3.4 | 100 | 60 | 0 | 0 | 0 | 0 |
| Rat 007 | Unilateral | Rehab alone | 92.5 | 3.4 | 100 | 75 | 0 | 0 | 0 | 0 |
| Rat 008 | Unilateral | Rehab alone | 80 | 3.4 | 100 | 72.5 | 0 | 0 | 0 | 0 |
| Rat 018 | Unilateral | Top 50% CLV | 70 | 3.4 | 100 | 70 | 0 | 0 | 0 | 0 |
| Rat 019 | Unilateral | Top 50% CLV | 55 | 3.4 | 100 | 60 | 0 | 0 | 0 | 0 |
| Rat 020 | Unilateral | Top 50% CLV | 55 | 3.4 | 100 | 47.5 | 0 | 0 | 0 | 0 |
| Rat 021 | Unilateral | Top 50% CLV | 52.5 | 3.4 | 100 | 80 | 0 | 0 | 0 | 0 |
| Rat 022 | Unilateral | Top 50% CLV | 85 | 3.4 | 100 | 70 | 0 | 0 | 0 | 0 |
| Rat 023 | Unilateral | Top 50% CLV | 62.5 | 3.4 | 100 | 67.5 | 0 | 0 | 0 | 0 |
| Rat 024 | Unilateral | Top 50% CLV | 92.5 | 3.4 | 100 | 80 | 0 | 0 | 0 | 0 |
| Rat 025 | Unilateral | Top 50% CLV | 87.5 | 3.4 | 100 | 82.5 | 0 | 0 | 0 | 0 |
| Rat 031 | Bilateral | Rehab alone | 92.5 | 96.6 | 0 | 50 | 75 | 97.1 | 22.5 | 47.5 |
| Rat 032 | Bilateral | Rehab alone | 90 | 97.9 | 37.5 | 52.5 | 85 | 97.9 | 37.5 | 40 |
| Rat 033 | Bilateral | Rehab alone | 85 | 97.6 | 40 | 40 | 95 | 97.8 | 67.5 | 75 |
| Rat 034 | Bilateral | Rehab alone | 85 | 98.3 | 45 | 57.5 | 82.5 | 97.3 | 25 | 35 |
| Rat 035 | Bilateral | Rehab alone | 80 | 96.8 | 5 | 12.5 | 85 | 97 | 15 | 30 |
| Rat 040 | Bilateral | Top 50% CLV | 85 | 97.6 | 25 | 57.5 | 82.5 | 97 | 15 | 62.5 |
| Rat 041 | Bilateral | Top 50% CLV | 72.5 | 97 | 7.5 | 40 | 82.5 | 98.7 | 82.5 | 45 |
| Rat 043 | Bilateral | Top 50% CLV | 90 | 96.8 | 5 | 37.5 | 92.5 | 96.8 | 0 | 42.5 |
| Rat 044 | Bilateral | Top 50% CLV | 82.5 | 96.8 | 5 | 20 | 82.5 | 96.6 | 5 | 20 |
| Rat 046 | Bilateral | Top 50% CLV | 95 | 99.7 | 45 | 80 | 95 | 100 | 62.5 | 77.5 |
